# Universal Truths about Reducing Meat Consumption?

**DOI:** 10.1007/s40572-025-00498-3

**Published:** 2025-09-18

**Authors:** Tatjana Kwasny, Sarah Marth, Karin Dobernig, Petra Riefler

**Affiliations:** 1 Institute of Marketing, University of Applied Sciences Wiener Neustadt, Schloegelgasse 22-26, Wiener Neustadt, 2700 Austria; 2 Institute of Marketing & Innovation, Department of Economics and Social Sciences, BOKU University, Feistmantelstrasse 4, Vienna, 1180 Austria; 3 Institute for Sustainability, University of Applied Sciences Wiener Neustadt, Zeiselgraben 4, Wieselburg, 3250 Austria

**Keywords:** Meat reduction, Meat consumption, Behavior change, Interventions, Global, Regional

## Abstract

**Purpose of Review:**

Current high levels of meat production and consumption are linked to severe health and environmental issues around the world. One remedy to this situation is a shift towards more plant-based diets. Several factors influence consumers’ meat intake including personal, socio-cultural, and economic factors as well as characteristics of the food environment. An increasing number of intervention studies are addressing these factors to support the necessary diet shift. However, the relative impact of factors on diets as well as the effectiveness of interventions might differ across geographic settings. This literature review describes (i) how far extant intervention studies on meat consumption reduction take the regional context into account, and (ii) whether current findings allow to distill universal factors that effectively reduce meat consumption across regions and countries.

**Recent Findings:**

This review identifies three key findings: First, existing evidence is largely limited to Europe and North America, while rapidly developing regions such as Africa and Asia are neglected despite rising meat consumption. Second, knowledge-related interventions appear to be more effective when framed around health rather than environmental issues of meat consumption. However, cross-country evidence on which specific issues matter most (e.g. cancer, cardiovascular disease etc.) is lacking. Moreover, social norm interventions seem to be effective in reducing meat consumption across countries, but research comparing effective designs and reference groups across countries is missing. Third, most interventions adopt standardized approaches with limited regional adaptation, except social norm interventions, which often tailor messages to the regional context.

**Summary:**

Due to a lack of cross-national studies, empirical evidence for universal factors spanning different regions or countries is scant. Also, whether the relevance of personal, socio-cultural or external factors varies in different regional contexts remains largely unclear. Thus, future research should emphasize the regional context and test adapted interventions in cross-country studies.

**Supplementary Information:**

The online version contains supplementary material available at 10.1007/s40572-025-00498-3.

## Introduction

Global meat production at current scales poses severe environmental challenges including deforestation and land degradation [1], biodiversity loss, water pollution, and the release of particularly potent greenhouse gas emissions such as methane [2]. In the context of climate change, reduced meat consumption has been recognized as a crucial strategy to lower greenhouse gas emissions [3]. To mitigate environmental problems, it is paramount to also address the demand-side and encourage a shift to plant-based diets and reduced meat consumption, especially in industrialized countries. From a public health perspective, a decrease in the consumption of red and processed meat can significantly lower the risk of noncommunicable diseases (NCD), including some cancers, cardiovascular diseases, and diabetes [4].

Meat consumption is driven by a complex interplay of economic, personal, and socio-cultural factors as well as characteristics in the food environment itself. For example, rising household income - particularly in developing countries - is strongly associated with higher meat consumption [5], as are urbanization, and women´s participation in the labor market [6]. Meat consumption has for a long time also been a marker of affluence and social status [7], and is associated with a „modern“ Western lifestyle [5]. Culture also plays a strong role in shaping dietary preferences, including meat consumption, as it is intertwined with social identity and status, and often deeply embedded in cultural traditions and rituals across different societies [8]. Meat is a main component in many traditional cuisines that define national or regional identity and is often central to religious rules [9].

Thus, efforts to reduce meat consumption might be more effective when taking regional consumption contexts into account. Similarly, the effectiveness of specific interventions might differ across geographic settings so that different interventions might be useful in different regions of the world. In this light, this literature review discusses (i) how far regional contexts are reflected in the design of extant intervention studies on meat consumption reduction, and (ii) whether current findings allow us to identify universal factors that effectively reduce meat consumption across regions and countries.

For this purpose, this literature review examines the key factors that affect a reduction in meat consumption and discusses how far these can be considered universal factors across different geographical contexts. It builds upon a recent systematic literature review of intervention studies aiming at meat consumption reduction [10]. The review’s authors developed the Meat Reduction Intervention Framework which categorizes interventions based on the personal, socio-cultural and external factors they target. We aim to expand this review by analyzing these factors from a geographic perspective. In the following sections, we first describe how we obtained, selected and analyzed our pool of studies. Second, we give an overview of intervention studies on reducing meat consumption across geographic regions. Third, we elaborate on geographical differences in the design of interventions relating to personal factors, socio-cultural factors, and characteristics in the food environment. Finally, we draw conclusions about universal truths about meat consumption and provide directions for future research to advance knowledge on the geographical relevance of meat reduction interventions.

## Method

For this review on the one hand, we included 83 from the 99 experimental studies identified by Kwasny et al. (2022) published between 2001 and 2019 [10]. The 16 studies we excluded tested interventions targeting long-term skill development and did not focus exclusively on dietary behavior, but also on broader health-related skills including smoking or physical activity. On the other hand, we added relevant studies published between 2020 and the end of 2023 using identical search strings and in/exclusion criteria [10] in Web of Science, Science Direct and SCOPUS. After removing duplicates and non-relevant studies based on the in/exclusion criteria, this systematic literature search yielded an additional set of 125 recently published experimental studies, which demonstrates the rapid development of this research field. In sum, our review thus included a final sample size of *N* = 208 studies. Two authors independently coded the studies using the MAXQDA software for qualitative data analysis. Our coding scheme assessed the focal factors of behavioral change (i.e. knowledge, emotions & cognitive dissonance, values & attitudes, social norms, and food environment), the framing of the study (environmental, animal welfare, health, or a combination), and specific study characteristics relevant for our analysis (i.e. geographic context, single country vs. cross-country design, and the degree of intervention adaptation). To examine the intervention content and degree of intervention adaptation, we further coded the experimental stimulus material (e.g., text-based versus visual information provided to participants). Finally, we synthesized the data by applying a thematic analysis and compiling a summary table that provides an overview of regional differences in intervention designs.

### Review of Intervention Studies on Reducing Meat Consumption with Focus on Geographical Context Sensitivity

Regarding intervention design, most studies of our study pool (*N* = 175) inform participants about the negative effects of meat consumption on the environment, animals or health. From those studies (see Table 1), most information measures refer to either the ecological footprint of meat consumption or animal welfare. Fewer address health aspects or combine two or more of those issues. There is a clear tendency to use standardized interventions without establishing any reference to the geographic context, while studies adapting their interventions to regional specificities are scarce. Turning to the geographical setting of data collection, as in many research areas, studies are mainly conducted in Europe and North America, whereas other regions are neglected despite their raising meat consumption levels (such as Africa and Asia) [11]. Finally, a vast majority of 198 studies collect data in a single country while studies engaging in cross-country comparisons are scarce.

**Table 1:** Overview of study characteristics and designs.

Characteristic	Sample Size (N)
**Information Frame**	
Environment	59
Animal Welfare	55
Health	38
Combinations	23
**Degree of Intervention Adaptation**	
standardized	165
adapted	32
standardized and adapted	2
**Geographical Spread**	
Europe	104
North America	92
Australia and Oceania	9
Asia	5
South America	3
**Country Setting**	
Single Country	198
Cross-Country	10

Note. In some cases, sample sizes within a category do not add up to the total number of studies included. This discrepancy arises from either double counting (for example, cross-country studies that are counted in multiple geographical regions) or missing information in individual studies, which results in those studies being excluded from the count.

We structure the analysis following the Meat Reduction Intervention Framework [10] categorizing interventions based on the personal, socio-cultural and external factors they address. In Table 2, the analyzed studies are categorized along with the factors addressed in the respective interventions (see columns), the region of data collection as well as the degree of intervention adaptation to the regional context (see lines). As a general finding, extant studies unevenly spread across the factors with a predominant focus on examining the impact of creating the food environment (64 studies) and overcoming knowledge gaps (71 studies) on meat consumption. Relatively few studies directly address dietary habits (11 studies) or consumer values (7 studies) in interventions (see Table 2).

**Table 2:** Distribution of focal factors of behavioral change per region and level of intervention adaptation.

Region	**Level of Intervention Adaptation**	Focal Factors of Behavioral Change							
		Overcoming Knowledge Gaps	Activating Emotions	Inducing Cognitive Dissonance	Addressing Consumer Values	Breaking Dietary Habits	Referring to Social Norms	Creating the Food Environment	Total
Europe	standardized	22	6	6	0	6	6	45	**91**
	adapted	8	2	0	0	1	7	0	**18**
	standardized and adapted	1	0	0	0	0	0	0	**1**
	unknown	3	0	0	1	0	0	1	**5**
	**Total**	**34**	**8**	**6**	**1**	**7**	**13**	**46**	**115**
North America	standardized	21	9	21	4	2	6	15	**78**
	adapted	6	2	0	0	0	9	0	**17**
	standardized and adapted	2	0	0	0	0	1	0	**3**
	unknown	5	0	0	0	0	0	0	**5**
	**Total**	**34**	**11**	**21**	**4**	**2**	**16**	**15**	**103**
Australia and Oceania	standardized	2	1	0	1	1	0	1	**6**
	adapted	0	0	1	1	1	0	0	**3**
	standardized and adapted	0	0	0	0	0	0	0	**0**
	unknown	0	0	0	0	0	0	0	**0**
	**Total**	**2**	**1**	**1**	**2**	**2**	**0**	**1**	**9**
Asia	standardized	0	0	2	0	0	0	2	**4**
	adapted	0	0	0	0	0	0	0	**0**
	standardized and adapted	1	0	0	0	0	0	0	**1**
	unknown	0	0	0	0	0	0	0	**0**
	**Total**	**1**	**0**	**2**	**0**	**0**	**0**	**2**	**5**
South America	standardized	0	2	1	0	0	0	0	**3**
	adapted	0	0	0	0	0	0	0	**0**
	standardized and adapted	0	0	0	0	0	0	0	**0**
	unknown	0	0	0	0	0	0	0	**0**
	**Total**	**0**	**2**	**1**	**0**	**0**	**0**	**0**	**3**

Note. In some cases, sample sizes within a category do not add up to the total number of studies included. This discrepancy arises from either double counting (for example, cross-country studies that are counted in multiple geographical regions or studies addressing multiple factors) or missing information in individual studies, which results in those studies being excluded from the count

In the following, we analyze studies for each intervention factor in more detail. For each factor, we will first provide some background information on the relevance of the factor in reducing consumers’ meat consumption to show the bigger picture to uninitiated readers. Subsequently, we will report details from our sample pool relating to our focal research questions, i.e. (i) how far regional contexts are reflected in the design of extant intervention studies on meat consumption reduction, and (ii) whether current findings allow us to identify universal factors that effectively reduce meat consumption across regions and countries.

### Overcoming Knowledge Gaps

Consumers´ lack of knowledge on the negative consequences of meat consumption on the environment, animal welfare or individual health acts as a barrier to reduce meat-eating [12]. Although information provision does not necessarily result in action, it appears to be a necessary precondition for behavioral change and helps consumers commit to reducing their meat consumption [13]. In other words, problem awareness is necessary to make consumers potentially ready for change.

Geographically, most studies testing the effect of knowledge through information provision are conducted in Europe and North America, whereas studies in other regions such as Australia and Oceania, Asia and South America are scarce. With regards to the information content provided, 25 studies in Europe highlight health issues related to meat consumption [e.g. 14], 25 studies present information about the environment [e.g. 15], while nine interventions emphasize animal welfare issues [e.g. 16]. Comparingly, in North America a majority of 26 studies focus on the environment [e.g. 17], while 19 interventions address health issues [e.g. 18] and 15 studies provide information about animal welfare [e.g. 19].

Information provided to consumers about the negative consequences of meat consumption is predominantly standardized (see Table 2), using information cues such as *“A large body of scientific evidence has shown that [consumption of meat and fish products …] has a negative effect on the climate worldwide.”* [20, p.4]]. A minority of 17 studies adapt such information to the respective country [e.g. 21, 22]. A few studies go further in adapting messages to the geographic context. As such, two studies in Norway adapt the information by showing regional people (i.e. typical Norwegians) explaining why they are reducing their meat consumption [23].

The current literature body on overcoming knowledge gaps shows a neglect to adapt information provision to geographic contexts. According to the theory of psychological distance, however, consumers perceive an object or event as more concrete when they feel psychologically close, but as more abstract when they feel psychologically distant. This is particularly relevant for climate change communication, since environmental issues are often perceived as psychologically distant problems that influence geographically distant people and places [24]. In this light, research on the relative effectiveness of adapted information interventions appears of relevance. Individuals might respond differently to messages about the consequences of food consumption adapted to regional contexts involving place-based narratives and stories linked to consumers’ everyday experiences compared to standardized information and mere facts [25].

Moreover, the body of extant studies suggests that information related to health issues has a stronger effect on intentions to reduce meat eating than information about environmental impacts [10]. One possible reason for this pattern is that egoistic motivations such as maintaining personal health tend to have a greater influence on behavior than altruistic motivations [26]. As cross-cultural studies comparing the effectiveness of health versus environmental frame interventions are lacking, it is unclear whether this pattern is of universal nature in the context of meat reduction.

### Activating Emotions

Emotions play a crucial role in meat consumption, across different countries and cultures [27]. The American Psychological Association (2024) describes emotions as “conscious mental reactions (such as anger or fear) subjectively experienced as strong feelings, usually directed toward a specific object and typically accompanied by physiological and behavioral changes in the body” [28]. Emotions such as joy and sadness are part of our everyday lives and influence eating behavior [29]. Information about meat can be provided to consumers in a way to shape their emotions and consequently influence their meat consumption [27]. Indeed, emotionally framed information seems to be more effective in reducing meat consumption than factually framed information [e.g. 21, 30]. Especially activating negative emotions around meat eating such as fear, guilt, sadness or disgust show to be effective in reducing intentions to eat meat [10]. Although less is known about the impact of positive emotions on meat consumption, there is some evidence that positive emotions can increase the intention to change towards more environmentally friendly behavior [e.g. 31, 32].

In our study pool, research on activating emotions primarily centers on eliciting negative feelings regarding meat eating. In North America, 11 studies focus on emotions aiming to activate negative emotions such as guilt, disgust, anger, fear, sadness or shame related to animal harm [e.g. 33]. In the European study pool, eight studies address emotions predominantly aiming to evoke feelings of disgust by, for example, providing information about health risks of contaminated meat [e.g. 21, 34]. Furthermore, one study aims to activate feelings of regret for not protecting health [35], while another study raises empathetic concern regarding animal welfare [36].

No studies compare the effects of emotionally and factually framed information across countries. However, there is evidence that although the antecedents of certain emotions are universally similar, the actual events activating certain emotions differ between cultures, e.g. perceived threats to individuals’ relationships antecede jealousy in many countries, while the actual events representing a threat differ [37]. Thus, the activation of emotions might lead to varying consumer responses and thus differently impact behavioral change in different regional contexts.

### Inducing Cognitive Dissonance

Emotions such as disgust or empathy for killed animals can activate meat-related cognitive dissonance among consumers. Cognitive dissonance describes a conflicting state of mind when a meat-eater’s behavior is inconsistent with her values, beliefs or attitudes. Consumers usually strive towards reducing this discomfort by applying strategies such as ignorance and denial of information or, ideally, by changing their actual (eating) behavior [38]. Activating consumers’ meat-related cognitive dissonance as well as overcoming strategies of dissonance reduction can foster dietary change towards reduced meat consumption [e.g. 39].

As Table 2 shows, there is a geographical divide in the research interest on inducing cognitive dissonance. In North America, 21 studies address cognitive dissonance while in Europe only six studies focus on this phenomenon. In our study pool, across countries, respondents’ cognitive dissonance is mainly activated by interventions reducing the dissociation between meat and its animal origin, for example by showing pictures of animals next to the meat dish [e.g. 39, 40]. Other interventions portray human-animal friendships to hinder consumers from dehumanizing animals and trigger cognitive dissonance [e.g. 41]. While this line of research focuses on animal-meat associations, cognitive dissonance may also be experienced over environmental or health concerns related to meat consumption. However, except for one study which activates cognitive dissonance by showing environmental information relating to greenhouse gas emissions of food products [42], current research fails to examine the relevance of health or environmental triggers to induce consumers’ meat-related cognitive dissonance.

Interventions aiming to reduce consumers’ dissociation between meat and its animal origin seem to particularly influence consumers’ willingness to eat meat in countries where typical consumers are rarely exposed to unprocessed meat (e.g. whole raw chicken). Such interventions aim to render participants more sensitive to information linking meat to living animals [43]. In a cross-country study, residents from the USA exposed to an image including the head of the animal experienced higher levels of disgust and empathy and were less willing to eat meat than Ecuadorian participants receiving the identical manipulation [43]. In a different cross-country study, French participants - but not Chinese participants - tended to deny animal mind. This means they attributed fewer human-like mental states to cows after being exposed to the animal’s origin. This finding hints to a psychological mechanism in place to reduce cognitive dissonance. However, independent of consumers’ regional context, they reduced their willingness to eat meat after highlighting the animal origin [44]. These results indicate that consumers across countries react differently to animal-meat associations and apply different strategies to reduce their cognitive dissonance. In this respect, interventions should be adapted to regional contexts to effectively change meat consumption through cognitive dissonance activation.

### Addressing Consumer Values

Values, such as universalism and self-direction [45] act as guiding principles of people’s behavior across life domains [46]. For example, Schwartz’ Theory of Basic Values postulates ten cross-cultural values structured along four dimensions: Openness to change (readiness for new developments), Self-Enhancement (striving for personal gain and comparative achievement), Self-Transcendence (care for the well-being and interests of others) and Conservation (importance of order, self-discipline and retention of traditions) [45]. Values have been shown to influence consumers’ behavior and consumption choices [47]. Consequently, values play a central role in shaping how consumers respond to information about reducing meat consumption. In particular, universalism - which emphasizes the welfare of society, nature, and the environment [45] - encompasses moral values such as animal welfare. High levels of these values, combined with the desire to maintain a moral self-image while continuing to eat meat, can result in ethical conflicts [48].

In light of the above, values appear relevant for inducing meat intake reductions. However, the number of experimental intervention studies addressing consumers’ higher order values are rare (see Table 2). Four out of six relevant studies focus on animal welfare and reference participants’ moral values [48, 49], aiming to shape beliefs about the wrongfulness of eating meat. For example, they achieve this by showing pigs and dogs in slaughterhouses and comparing them along morally relevant dimensions [48]. One other study refers to consumers’ self-transcendence or self-enhancement values by communicating the environmental consequences of meat-eating [50]. Moreover, one intervention focuses on hierarchy and dominance values of consumers by communicating the values symbolized by meat [51]. Other topics such as activating values related to health are not addressed by existing studies.

From the few studies addressing consumers’ values, all but one study applies a standardized intervention without any reference to the study setting. An exemplary exception is a study in New Zealand which tests an intervention referring to the negative environmental impact of meat consumption on New Zealand [50]. Thus, existing research does not provide insights into whether standardized or adapted value interventions are more effective in changing meat-eating behaviors. Considering the lack of cross-country studies, it is unclear whether interventions referring to participants’ values differ in their effectiveness based on the geographic setting and/or the value addressed.

### Breaking Dietary Habits

Habit is an automatic reaction to cues that prompts behavior quickly and with minimal thought, often bypassing conscious intentions. Habits endure even when motivation wanes, making them a valuable mechanism for long-term behavior change interventions [52]. Consumers’ habits influence their meat consumption [12] in that consumers tend to stick to eating routines and automatically choose meat in certain contexts (e.g. when eating at restaurants) [53]. Kollmuss and Agyeman (2002) hypothesize that even when people are eager to change their behavior, they might not succeed until the new behavior is practiced long enough and becomes a habit [54]. Despite the central role of habits in influencing meat consumption, this factor of behavioral change is scarcely addressed in existing experimental research. However, there is some evidence that pledging (i.e. making a commitment to behave in a certain manner) and goal setting (i.e. setting goals to change one’s eating behavior and concrete implementation plans how to do so) support breaking consumers’ dietary habits. More specifically, consumers who commit to reducing their meat consumption for a certain period beforehand tend to report reduced levels of meat consumption [e.g. 53, 55, 56].

The effectiveness of pledging and self-commitment strategies in reducing meat consumption seems to depend on certain place-based factors. In one comparative study across the UK, Germany and Australia, all participants show lower self-reported meat consumption over 28 days after they have pledged to do so. German participants, however, show significantly higher reduction rates than participants from the other two countries [56]. The authors attribute this difference to greater variety of meat-less options available in German supermarkets which supports German consumers in their attempts to reduce their meat consumption [56, 57].

Relating to the intervention design, out of 11 studies focusing on consumers’ habits, only two studies provide adapted information about regional health and environmental impacts of meat consumption to participants [55, 58]. Given the limited scope of adapted approaches, it remains unclear how interventions can be optimally designed to more effectively change dietary habits within specific regional contexts. Interventions targeting habit change could potentially be enhanced by tailoring reduction goals to align with regional consumption patterns - for example, by referring to locally available and popular meat alternatives.

### Referring To Social Norms

Social norms serve as overarching guidelines for behaviors and attitudes within social groups. They are often conveyed through information about the typical actions of others within a relevant social group [59, 60]. Adhering to these norms enables individuals to align themselves with and feel connected to the group [61, 62]. In most societies, eating meat is still perceived as a social norm. Additionally, it allows consumers to express their national social identity, for instance through food traditions such as having Sunday roasts in the UK [63] or hot dogs in the USA [64].

In our study pool, intervention studies mostly refer to descriptive norms, which indicate how common behavior is in a relevant social group. They do so by either highlighting the current state of behavior (e.g. the percentage of consumers currently limiting their meat consumption) or an ongoing change (e.g. the percentage of consumers who have recently started trying vegetarian diets) [e.g. 65–68]. Generally, social norm messages seem to be effective in reducing intentions to eat meat, especially when reduced meat consumption is framed as ongoing change [e.g. 69]. Moreover, research shows that the presence of vegetarians increases the likelihood of choosing a vegetarian dish [70].

In stark contrast to other intervention types, social norm interventions are frequently adapted to the specific country of participants (see Table 2). Interventions read, for example, as follows: “*Recent research by the Voedingscentrum (2022) has shown that around 80% (majority)/20% (minority) of the Dutch population is either trying*,* or considering to make an effort to limit the amount of meat they consume…*” [63, p. 6]]. This tendency suggests some implicit assumption that social norms are particularly effective when related to other citizens in the country [72]. However, research explicitly comparing the effectiveness of such adaptation relative to standardized norm messages (e.g., *“We’ve noticed customers are starting to choose more meatless dishes.”* [68, p. 7]]) is mostly missing. A single study in our pool directly compares these two types of norm messages with reference to climate impacts on intentions to reduce meat eating [73]. Contrary to expectations, in this study standardized social norm messages are found to be more effective than social norm messages referring to the country [73]. The authors conclude that adapted norm interventions referring to reduced meat consumption of citizens in the home region might decrease participants’ perceived need for action to mitigate climate impacts. The underlying mechanism might be an optimism bias, meaning that consumers tend to discount personal risk to a given situation or event and hold optimistic views towards their personal locations and their residents [73–75]. In light of the limited number of studies, there is no evidence to determine whether social norm messages tailored to a specific nationality are more effective at reducing meat consumption than standardized norm messages.

Turning to cross-country differences in the effectiveness of norm interventions, the overall picture is similar to the above. Only one study in our pool examines responses to social norm messages in a cross-national setting among participants from the Netherlands and Germany [71]. This study reports no significant differences between the two countries.

### Creating the Food Environment

Meat consumers can also be approached in the food environment where they eat meat, i.e. in restaurants [e.g. 76], canteens [e.g. 77] or grocery stores [e.g. 78]. Here, interventions aim to explicitly support consumer choice as well as facilitate automatic decision making by nudging them towards more sustainable food choices [79].

Of these interventions in the food environment, studies were predominantly conducted in Europe, with only a few studies in North America and China (see Table 2). Out of the 46 studies in Europe, 29 interventions promote vegetarian food choices by making them more visible or appealing to consumers. For example, vegetarian food is placed more prominently in stores and buffet lines [e.g. 80, 81], is visually highlighted on the menu [e.g. 82] or promoted as the dish of the day [e.g. 83]. Six studies highlight vegetarian dishes on the menu either by making them the default option [e.g. 84] or using more appealing labels [e.g. 85]. The remaining 11 interventions in Europe change the portion sizes or the price of vegetarian foods [e.g. 76, 86]. Comparingly, in North America only two interventions enhance the visibility of vegetarian foods [87, 88], but seven interventions offer vegetarian alternatives by default to increase the effort to choose the non-default meat option [e.g. 89] and six studies increase the portion sizes of vegetables for meat dishes [e.g. 90]. In China, two intervention studies present vegetarian food with varying background colors in different virtual reality restaurant settings [91, 92]. Across all geographic regions, study design mainly offers raw or cooked vegetables or typical vegetable dishes (e.g. vegetable pasta or patties) as meat alternatives [e.g. 85, 91, 93]. In Europe, some studies offer meat substitutes such as plant-based sausages [e.g. 78].

With regards to cross-country studies, there was some more effort in comparing the effectiveness of food environment interventions across geographical context. Specifically, four studies assess cross-country differences among European consumers [94–96], with only one reporting significant differences between countries. This study shows that offering a vegetarian dish by default significantly increases vegetarian choices, with Dutch consumers being more likely to choose vegetarian options than Danish consumers. However, this finding might be explained by the larger share of meat-reducers (i.e. individuals who have already started to reduce their meat consumption and thus, are potentially more responsive to interventions) in the Dutch sample [94]. Based on the limited number of cross-country studies, evidence on which types of interventions are effective in the food environment in certain geographic regions is lacking. However, food and dish preferences are strongly influenced by culture and regional contexts [97], thus, the way of presenting food and the type of vegetarian alternatives offered in the food environment might impact consumers’ choices differently.

## Conclusion, Limitations and Future Research Directions

The number of experimental studies examining the effectiveness of interventions for reducing consumers’ meat consumption is rapidly growing. Since food - and meat consumption in particular - is deeply embedded in local cultures and traditions, carrying different meanings across countries, this review aims to investigate the extent to which current research in this field considers geographic contexts. This review draws three main conclusions regarding this question.

First, existing findings predominantly originate from Europe and North America, both regions characterized by high levels of per capita meat consumption. This focus is self-evident, as these regions have a pressing need to reduce meat consumption to address environmental and health concerns. However, it will become increasingly relevant to promote more plant-based diets also in developing regions such as Africa and Asia where the growth in meat consumption is projected to be the highest. Thus, it seems necessary to identify the key factors influencing meat consumption that are particularly relevant to these regional contexts and to design effective interventions accordingly. Second, despite scattered research across countries and limited efforts in cross-country comparisons, we find some evidence suggesting which interventions may be generally effective in reducing meat consumption and potentially hold universal relevance. Given the available evidence, when aiming to use knowledge-related interventions, addressing health-related topics associated with meat consumption appears to be more effective than providing environmental information in reducing intentions to eat meat. However, due to a lack of cross-country studies, it remains unclear which specific health issues (e.g. cancer, cardiovascular disease etc.) or environmental issues (e.g. climate change, pollution, biodiversity loss etc.) linked to meat consumption [98, 99] are most relevant in specific geographic regions. Further, information appears to be even more effective when framed emotionally, particularly eliciting negative emotions toward meat consumption. Consumers with different cultural backgrounds might react differently to such messages, however, research on the role of emotions in reducing meat consumption across countries is lacking. Furthermore, information linking meat to its animal origin shows to be generally effective in reducing intentions to eat meat by activating cognitive dissonance. However, consumers across countries react differently to information highlighting animal-meat associations and seemingly use different strategies to overcome their dissonance. Consequently, more comparative research in this field is needed.

Regarding interventions addressing other factors than knowledge and emotions, social norm interventions appear to be effective in reducing meat consumption across countries. More detailed comparisons of effective designs and reference groups are currently missing and needed. Further, changing the food environment such as restaurants to foster behavioral change towards vegetarian food appears promising in changing behavior in Europe and North America. However, evidence from cross-country studies is limited, thus it is not clear which types of vegetarian dishes should be promoted in different regional contexts and how interventions should be designed to effectively promote these meat alternatives. Third, existing intervention studies mainly provide consumers with standardized interventions, while adapting information to the regional context (e.g. in terms of narratives, language etc.) could potentially increase message effectiveness. Social norm interventions represent an exception in this regard, as they typically tailor their norm messages to the focal country. Beyond these, studies that explicitly consider geographical contexts and their specificities remain scarce. A limited number of studies compare the effectiveness of standardized versus adapted interventions, but their findings have not yet yielded clear guidance for future research and policymakers.

Our review comes with some limitations. All included studies are experimental in nature, which means we may have overlooked other cross-regional studies that offer insights into universal factors influencing meat consumption reduction. Additionally, our analysis is restricted to studies published in English, potentially excluding relevant research conducted in other regions, such as Asia.

To conclude, existing intervention studies address personal factors, socio-cultural factors and external factors such as characteristics in the food environment, which are all assumed to play a crucial role in meat consumption behavior. Only a few existing studies test interventions in cross-country settings. Thus, whether universals truths regarding the reduction of meat consumption exist, is still unclear as to date, it remains unknown which factors are most relevant in specific regional contexts. Future research could shed light on this by testing more adapted interventions in cross-country study settings and potentially identifying universal factors that reduce meat consumption across regions and countries including regions that are currently under-researched.

## Electronic Supplementary Material

Below is the link to the electronic supplementary material.

Supplementary Material 1

## Data Availability

No datasets were generated or analysed during the current study.

## Key References

- Kwasny T, Dobernig K, Riefler P. Towards reduced meat consumption: A systematic literature review of intervention effectiveness, 2001–2019. Appetite. 2022 Jan 1;168. **Our review builds on this systematic literature review of interventions and on the factors identified in the Meat Reduction Intervention Framework.**
- Kunst JR, Palacios Haugestad CA. The effects of dissociation on willingness to eat meat are moderated by exposure to unprocessed meat: A cross-cultural demonstration. Appetite. 2018;120:356–66. **This paper is one of the few that identifies cross-cultural differences among consumers’ response to an intervention activating cognitive dissonance.**
- Tian Q, Hilton D, Becker M. Confronting the meat paradox in different cultural contexts: Reactions among Chinese and French participants. Appetite. 2016 Jan 1;96:187–94. **This paper is one of the few that identifies cross-cultural differences among consumers’ response to an intervention activating cognitive dissonance.**
- Piazza J, Gregson R, Kordoni A, Pfeiler TM, Ruby MB, Ellis DA, et al. Monitoring a meat-free pledge with smartphones: An experimental study. Appetite. 2022 Jan 1;168. **This paper is the only one identifying cross-cultural differences among consumers’ response to an intervention aiming to change habits.**
- Hielkema MH, Onwezen MC, Reinders MJ. Veg on the menu? Differences in menu design interventions to increase vegetarian food choice between meat-reducers and non-reducers. Food Qual Prefer. 2022 Dec 1;102. **This paper is the only one identifying cross-cultural differences among consumers’ response to an intervention implementing changes in the food environment (nudges).**
